# Frailty and decisional regret after elective noncardiac surgery: a multicentre prospective cohort study

**DOI:** 10.1016/j.bja.2024.08.001

**Published:** 2024-09-03

**Authors:** Yonathan Agung, Emily Hladkowicz, Laura Boland, Husein Moloo, Luke T. Lavallée, Manoj M. Lalu, Daniel I. McIsaac

**Affiliations:** 1 Faculty of Medicine, University of Ottawa, Ottawa, ON, Canada; 2 Ottawa Hospital Research Institute, Ottawa, ON, Canada; 3 Centre for Surveillance and Applied Research (CSAR), Public Health Agency of Canada, Ottawa, ON, Canada; 4 Department of Surgery, Division of General Surgery, University of Ottawa, Ottawa, ON, Canada; 5 Department of Surgery, Division of Urology, University of Ottawa, Ottawa, ON, Canada; 6 Departments of Anesthesiology and Pain Medicine, University of Ottawa and The Ottawa Hospital, Ottawa, ON, Canada; 7 School of Epidemiology and Public Health, University of Ottawa, Ottawa, ON, Canada

**Keywords:** comorbidity, decisional regret, disability, epidemiology, frailty, postoperative complications, surgery

## Abstract

**Background:**

Frailty is associated with morbidity and mortality after surgery. The association of frailty with decisional regret is poorly defined. Our objective was to estimate the association of preoperative frailty with decisional regret status in the year after surgery.

**Methods:**

We conducted a secondary analysis of a prospective, multicentre cohort study of patients aged ≥65 years who underwent elective noncardiac surgery. Decisional regret about having undergone surgery was ascertained at 30, 90, and 365 (primary time point) days after surgery using a 3-point ordinal scale. Bayesian ordinal logistic regression was used to estimate the association of frailty with decisional regret, adjusted for surgery type, age, sex, and mental health conditions. Subgroup and sensitivity analyses were conducted.

**Results:**

We identified 669 patients; 293 (43.8%) lived with frailty. At 365 days after surgery, the unadjusted odds ratio (OR) associating frailty with greater decisional regret was 2.21 (95% credible interval [CrI] 0.98–5.09; *P*(OR>1)=0.97), which was attenuated after confounder adjustment (adjusted OR 1.68, 95% CrI 0.84–3.36; *P*(OR>1)=0.93). Similar results were estimated at 30 and 90 days. Additional adjustment for baseline comorbidities and disability score substantially altered the OR at 365 days (0.89, 95% CrI 0.37–2.12; *P*(OR>1)=0.39). There was a high probability that surgery type was an effect modifier (non-orthopaedic: OR 1.90, 95% CrI 1.00–3.59; *P*(OR>1)=0.98); orthopaedic: OR 0.87, 95% CrI 0.41–1.91; *P*(OR>1)=0.36).

**Conclusions:**

Among older surgical patients, there appears to be a complex association with frailty and decisional regret, with substantial heterogeneity based on assumed causal pathways and surgery type. Future studies are required to untangle the complex interplay between these factors.

**Editor's key points**

• Frailty is now well recognised as a key determinant of health outcomes, including after surgery. It therefore seems likely that patients with frailty may be more likely to regret later their decision to undergo surgery.
• This study demonstrated that the association between frailty and decisional regret exists and identified a range of other factors that might explain this observation. Different types of surgical patients have different patterns of decisional regret.
• Orthopaedic surgery was associated with much less decisional regret, perhaps reflecting the low-risk nature of surgery such as joint replacement with good health outcomes, even among paients with frailty.
• A more sophisticated, individualised approach to shared decision-making is needed for frail patients. Blanket ‘risk prediction’ models covering all patient groups are unlikely to help.

Frailty is a multidimensional state resulting from the accumulation of age and disease-related deficits leaving individuals vulnerable to surgical stress.^1^^,^^2^ As our population ages, a growing number of individuals with frailty will present for surgery.^3^ Frailty is associated with a ≥2-fold increase in morbidity and mortality and a 5-fold increase in loss of independence.^4^^,^^5^ Although the high rate of adverse events experienced by older patients with frailty undergoing surgery is well established, the long-term implications of adverse outcomes on patients' postoperative trajectories remains unclear.

Decisional regret is defined as ‘a negative emotion involving distress or remorse following a decision’.^6^^,^^7^ Regret can occur after a treatment decision, such as having surgery.^6^ Data suggest that 6–24% of patients experience postoperative decisional regret.^8^ One reason that rates of decisional regret differ between studies is that different types of decisional regret exist, including regret focused on the *process* leading to a decision, the *option* chosen, or the *outcome* of the decision.^9^ As patients considering surgery often face a difficult choice between invasive treatments with relatively high short-term risks (e.g. having surgery) and more conservative options with uncertain longer-term risks (e.g. medical management or watchful waiting), option regret is a key focus for perioperative patients and researchers.

Although numerous preoperative predictors of decisional regret exist (e.g. depression, 5-fold increase; anxiety, 3-fold increase; female sex, 28% increase; postoperative complication, 2-fold increase),^10^, ^11^, ^12^, ^13^ the association between frailty and postoperative decisional regret remains largely unaddressed.^4^^,^^14^ To our knowledge, one single-centre study has estimated the association of frailty with postoperative decisional regret and was limited to predominantly male head and neck surgery patients with 30% missing outcome data.^15^ Although this study did not find a significant adjusted association between frailty and decisional regret, there remains an important need to evaluate the association between frailty and decisional regret in a more generalisable population of older surgical patients. Ideally, such an evaluation would include multicentre data and capture decisional regret at multiple time points,^7^ including up to a year after surgery, where rates of decisional regret may peak.^16^^,^^17^

Understanding whether frailty is associated with decisional regret across the first postoperative year could help inform patients in their surgical decision-making process and assist clinicians in providing older patients with optimal support, such as shared decision-making. Our objective was to estimate the association of preoperative frailty with decisional regret over the year after major elective surgery.

## Methods

### Design and setting

This was a secondary analysis of a prospective, multicentre cohort study conducted at three hospitals in the Canadian province of Ontario.^18^^,^^19^ Two centres were academic hospitals, and the third was a community-practice-oriented centre serving a primarily francophone population. A sub-study-specific protocol was registered.^20^

### Population

Older adults (aged ≥65 years) who underwent a planned (i.e. elective) inpatient noncardiac surgery (i.e. not involving the heart or need for cardiopulmonary bypass), who were able to communicate in English or French, and who expected to be available by telephone after surgery, consented in writing. Individuals who lacked the cognitive ability to answer outcome scales were not included (individuals with mild to moderate cognitive dysfunction were eligible). Recruitment occurred from September 1, 2015, to June 30, 2017. Ethical approval was granted (Ottawa Health Sciences Network–Research Ethics Board #20150342-01H; Hôpital Montfort Research Ethics Board #DM-31-08-15). This study is reported per appropriate guidelines.^21^^,^^22^

### Exposure

Our primary exposure was the presence of frailty, based on a Clinical Frailty Scale (CFS^2^) score of ≥4 (an optimal cut point among older surgical patients^19^) The CFS is a clinically oriented tool developed from the accumulating deficits conceptual model of frailty.^2^ Frailty assessments were conducted in the preoperative anaesthesiology clinic at each hospital by trained research assistants^23^ or the primary investigator (DIM). We also collected a specific CFS score for each participant to support sensitivity analyses.

### Outcomes

The outcome was decisional regret status about the option chosen to have surgery, which was collected in person (if an individual was hospitalised at follow-up) or by phone. As recommended, regret was measured at multiple time points (postoperative days 30, 90, and 365).^7^ Although no single measure of decisional regret is used consistently across studies, metrics range from lengthy questionnaires to simple binary questions. However, most studies present data based on dichotomisation (leading to information loss).^8^ Our patient partners encouraged the research team to minimise respondent burden from lengthy outcome scales, which is particularly impactful on older individuals.^24^ As such, our outcome metric was based on the Decisional Regret Scale's option regret item but worded specifically for the surgical decision, ‘If you could go back in time, would you still choose to have your surgery based on your experience up to now?’^6^^,^^9^ In validation studies, this item had the highest item–total correlation with overall decisional regret for surgical decisions.^6^ The item was answered on a 3-point ordinal scale (ordered as follows: no, unsure, and yes), acknowledging that this study-adapted approach has not been explicitly validated. The primary outcome point was 365 days after surgery (where rates of decisional regret may peak^16^^,^^17^); secondary outcomes were responses at 30 and 90 days after surgery.

### Covariates

Baseline data were collected, informed by best practice guidelines for preoperative assessment of older adults.^25^ These included recording age, sex, patient-reported comorbidities included in the Elixhauser set,^26^ cognitive status (AD8 score^27^), baseline disability score,^28^ and risk of depression or anxiety (PHQ-2^29^), along with the surgical specialty (orthopaedics [primarily total joint arthroplasty], abdominal [primarily general surgery, urology, and gynaecologic oncology cases], thoracic, vascular, and neurologic).

### Analysis

Descriptive characteristics were calculated by frailty status and compared between groups using absolute standardised differences.^30^ Data management was performed using SAS v9.4 (SAS Institute, Cary, NC, USA). Bayesian analyses were performed using the R statistical language (R Foundation for Statistical Computing, Vienna, Austria; packages ‘brms’ and ‘rmsb’^31^^,^^32^).

To estimate the association of preoperative frailty with decisional regret status 365 days after surgery, we calculated the unadjusted and adjusted odds ratios (ORs), 95% credible intervals (CrIs), and the probability of a non-null effect for each association (in this case, the probability that the OR was greater than 1; *P*(OR)>1) using Bayesian ordinal logistic regression. In this analysis, the OR can be interpreted as the relative odds of reporting a higher level of decisional regret status among those with frailty compared with those without. The 95% CrI can be interpreted as the range of values, based on prior knowledge and the data, that would have a 95% probability of containing the true value. As we had repeated outcome measures, all models included terms for exposure, time (categorical), exposure×time, and a random effect for each patient to account for the repeated measurement. For the adjusted primary analysis, we included terms for sex (binary), age (tensor product spline), surgical specialty (categorical), and presence of anxiety or depression risk (binary). These variables were identified as confounders, as clinical and epidemiologic knowledge suggest that they are likely to be associated with frailty and decisional regret and were understood to lie before frailty in the causal pathway.^10^, ^11^, ^12^, ^13^^,^^33^ For each model, we used weakly informative priors, which are recommended as they decrease the likelihood of considering extreme and unrealistic values without having substantial influence on estimated parameters (see Supplementary Appendix S1 for descriptions of Bayesian workflows, including prior distributions, and directed acyclic graphs).^34^ Point estimates were based on the median of the posterior distribution; CrIs and *P*(OR>1) were based on the highest density posterior interval.

### Sensitivity analyses

Assumptions of causal pathways to frailty (i.e. risk of overadjustment bias attributable to conceptual contributions of disability and comorbidity to frailty^3^^,^^33^^,^^35^^,^^36^) may differ based on the chosen conceptual definitions of frailty. Our exposure, the CFS, is based on the accumulating deficits model, where disability and comorbidity contribute to (i.e. precede) frailty.^37^ Under these assumptions, adjusting for disability and comorbidity would lead to overadjustment bias. However, the phenotype model considers disability and comorbidity to be distinct from frailty.^1^^,^^35^ Therefore, we conducted a sensitivity analysis where additional adjustment was included for the baseline number of comorbidities and disability score as linear terms. We also conducted effect modification analyses based on surgery type (orthopaedic *vs* non-orthopaedic, as a multiplicative interaction term between frailty status and an orthopaedic surgery indicator variable). Finally, we assessed frailty's association with decisional regret using a categorical parameterisation (≤3 (reference), 4, and ≥5) to explore the possible change in the odds of decisional regret that may exist with increasing levels of frailty.

### Sample size, power, and missing data

Although our analysis used Bayesian methods, we followed guidance from Harrell^38^ to inform an initial power estimate based on frequentist methods (as Bayesian power analyses typically involve complex simulations whose required parameters we did not possess). Based on the control group distribution of decisional regret responses (no = 0.9, unsure = 0.05, and yes = 0.05), we expected to have 80% power to detect an OR as low as 2.0 using a significance level of 5%.^39^

As some outcome data were missing (owing to nonresponse, death, or both, as a competing risk) we prespecified several approaches to missing data. For our primary analysis, we performed adjusted analyses using five multiply imputed datasets (simulation data suggest that five imputed data provide precision for the point estimate while allowing us to balance the computational demands of estimating Bayesian models).^40^ Imputation was performed under a missing-at-random assumption, using fully conditional specification and predictive mean matching to impute missing decisional regret responses (R package ‘mice’^41^; see Supplementary Appendix S2). The multiple imputation model was constructed using all available exposure, covariate, and outcome data, including disability scores and mortality events identified at each follow-up time point, as previously reported in primary reports from this cohort.^19^^,^^42^ Our posterior distributions represented the combined integration of posteriors from all imputed datasets, as combining results of imputed datasets fit directly within the Bayesian framework (in contrast to frequentist methods that rely on statistical rules). To test the robustness of our results, we also analysed our complete case data using repeated measures, which we would expect to be unbiased under a missing completely-at-random mechanism.^43^ We considered a 95% CrI excluding the null value, a *P*(OR>1)>95%, or both, as strong evidence of an association.

## Results

We identified 669 patients aged ≥65 years who underwent elective, major noncardiac surgery; 293 (43.8%) lived with preoperative frailty. The mean age was 73 (Range: 60–99) years, 317 (47.4%) were female, and 325 (48.6%) had orthopaedic surgery. Table 1 provides patient characteristics based on the presence of preoperative frailty. Patients with frailty were more likely to have co-existing comorbidity and baseline disability. Patients with frailty presented more often for orthopaedic surgeries, whereas patients without frailty presented more often for abdominal surgeries. Decisional regret responses were complete for 597 (89.2%), 598 (89.4%), and 579 (86.6%) patients at days 30, 90, and 365 after surgery, respectively (see distribution of baseline characteristics by missing data status and participant flow in Supplementary Appendix S2).

**Table 1:** Baseline characteristics by frailty status. ASD, absolute standardized difference; sd, standard deviation.

	Frailty		ASD
	No (*n*=376)	Yes (*n*=293)	
Patient characteristics			
Age (years), mean (sd)	73.1 (60-99)	74.4 (60-95)	0.21
Comorbidity, mean (sd)	2.1 (1.4)	2.5 (1.7)	0.26
Disability, mean (sd)	12.3 (11.4)	34.0 (14.1)	1.69
Female (%)	41.2	56.6	0.31
Cognitive dysfunction (%)	14.2	22	0.20
Depression (%)	3.5	14.2	0.38
Surgery (%)			
Abdominal	28.2	12.3	0.40
Neurologic	4.5	6.8	0.10
Orthopaedic	40.2	59.6	0.40
Thoracic	12.5	7.2	0.18
Vascular	14.6	14.3	0.01

### Decisional regret

**Fig 1:**
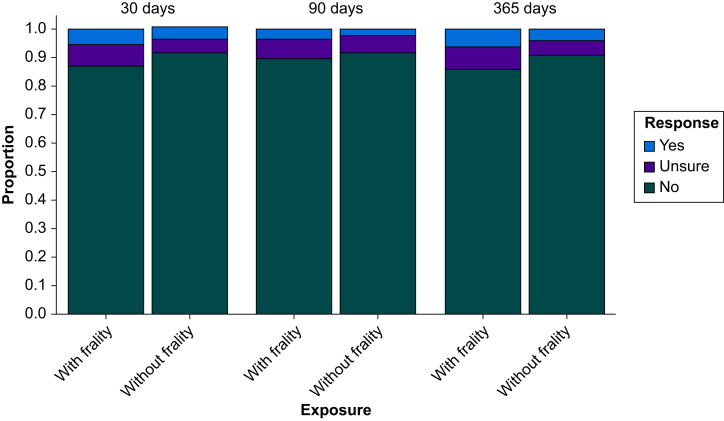
Proportion of patients with and without frailty experiencing different severities of decisional regret at each postoperative time point.

**Fig 2:**
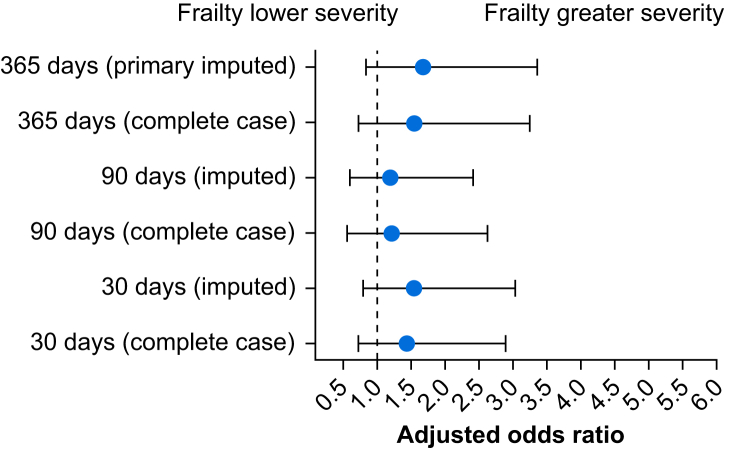
Forest plot of the adjusted association of frailty with decisional regret status at each time point from adjusted models that included terms for sex, age, surgical specialty, and presence of anxiety or depression risk. Imputed analyses used five datasets imputed using all available covariate and outcome data. Point estimates represent the median of the posterior distribution, and bars represent the 95% credible interval based on the highest density posterior interval.

The status of decisional regret with and without frailty is presented in Figure 1 (Supplementary Appendix S3). Decisional regret status was worse for those with frailty at each time point. Model diagnostics supported appropriate model fit and convergence (Supplementary Appendix S1). The odds of reporting higher decisional regret status at 365 days after surgery in patients with frailty compared with those without are outlined in Figure 2. The unadjusted OR for decisional regret at 365 days after surgery in patients with frailty compared with those without was 2.21 (95% CrI 0.98–5.09; *P*(OR>1)=0.97), and after adjustment, OR was 1.68 (95% CrI 0.84–3.36; *P*(OR>1)=0.93). The use of informative prior distributions resulted in similar estimates (Supplementary Appendix S4).

### Secondary and sensitivity analyses

Unadjusted and adjusted ORs for decisional regret status results were similar in direction and strength of association at 30 (unadjusted OR 2.13, 95% CrI 0.90–5.04; *P*(OR>1)=0.96; adjusted OR 1.56, 95% CrI 0.8–3.04; *P*(OR>1)=0.90) and 90 days after surgery (unadjusted OR 1.68, 95% CrI 0.7–4.1; *P*(OR>1)=0.88); adjusted OR 1.20, 95% CrI 0.6–2.42; *P*(OR>1)=0.70) (Fig. 2). The repeated-measures complete case adjusted analysis was similar in direction, magnitude, and precision to the multiply imputed primary analysis (Fig. 2).

**Fig 3:**
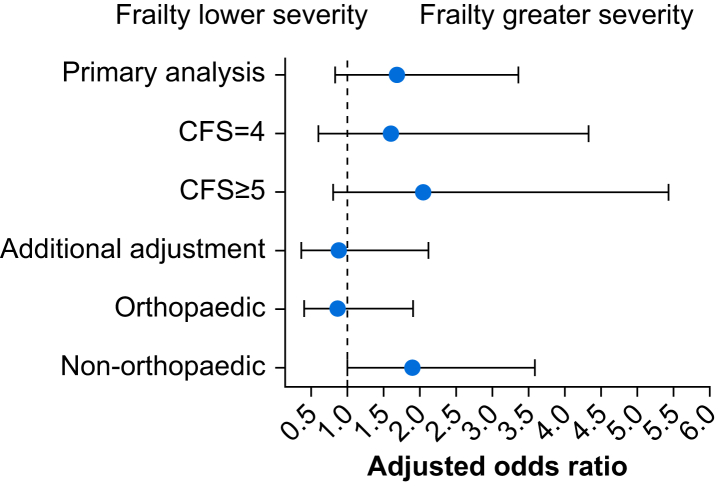
Forest plot of the adjusted association of frailty with decisional regret status in primary and sensitivity analyses. Unless specified, adjusted models included terms for sex, age, surgical specialty, and presence of anxiety or depression risk. ‘Additional adjustment’ added terms for baseline count of comorbidities and patient-reported disability score. Clinical Frailty Scale (CFS) analyses had scores 1–3 as reference. Point estimates represent the median of the posterior distribution, and bars represent the 95% credible interval based on the highest density posterior interval.

With additional adjustment for baseline comorbidity count and disability score, the adjusted frailty–decisional regret association was directionally reversed. Those with frailty experienced lower decisional regret status at 365 (OR 0.89, 95% CrI 0.37–2.12; *P*(OR>1)=0.39) (Fig. 3), 30 (OR 0.87, 95% CrI 0.40–1.93; *P*(OR>1)=0.36) and 90 days after surgery (OR 0.70, 95% CrI 0.29–1.70; *P*(OR>1)=0.21).

We also identified a 94% probability that the frailty–decisional regret association was modified by surgery type (Fig. 3). After non-orthopaedic surgeries, decisional regret status was greater for those with frailty (OR 1.90, 95% CrI 1.00–3.59; *P*(OR>1)=0.90), whereas after orthopaedic surgeries, those with frailty had lower decisional regret status (OR 0.87, 95% CrI 0.41–1.91; *P*(OR>1)=0.36).

There was some evidence of greater decisional regret status in those with higher CFS scores (Fig. 3). Compared with those without frailty, patients with a CFS of 4 had a 1.61-fold (95% CrI 0.61–4.33; *P*(OR>1)=0.83) increase in the odds of decisional regret status, whereas patients with a CFS ≥5 had a 2.06-fold (95% CrI 0.81–5.43; *P*(OR>1)=0.94) increase.

## Discussion

In this multicentre, prospective cohort study of older patients having elective, noncardiac surgery, we estimated high probabilities that older adults with frailty experience a greater likelihood of decisional regret across the first year after surgery than people without frailty. However, this association is complex and may be modified by the type of surgery an older adult is having and the choice of confounders that are adjusted for. After orthopaedic surgery, frailty was associated with lower likelihood of decisional regret, whereas after non-orthopaedic surgery, frailty was associated with greater likelihood. Furthermore, additional adjustment for baseline comorbidity and disability resulted in an estimate where frailty was associated with a lower likelihood of regret, suggesting that the complex interplay between the distinct but related^35^ concepts of frailty, comorbidity, and disability requires unique consideration to inform a patient's risk of postoperative decisional regret.

Preoperative frailty assessment is recommended by multiple clinical practice guidelines to inform shared decision-making, care planning, and preoperative optimisation.^44^^,^^45^ Because older adults with frailty are at >2-fold risk of short-term morbidity, mortality, and disability, many hypothesise that these individuals may be at greater risk of postoperative decisional regret that could indicate dissatisfaction with decision-making, surgical outcomes, or both.^5^^,^^46^ To our knowledge, frailty-specific data are limited to a cohort of 274 patients having head and neck surgery at a single centre, which found a significant unadjusted association (OR 1.38, 95% confidence interval [CI] 1.01–1.90 per unit increase in the Fried phenotype) between frailty and decisional regret, but no significant adjusted association (*P*>0.05; OR and CI not provided).^15^ In our study, the rates of decisional regret in older patients with and without frailty (∼10% with or unsure about regret) were comparable to rates of decisional regret reported across surgical patients (6–24%).^8^ Rates of decisional regret were also relatively stable over the year after surgery. However, although we found a high probability that the likelihood of decisional regret was higher in people with frailty on an unadjusted basis across time points, adjustment for prespecified confounders attenuated the effect size, decreased the probability of a non-null effect, and suggested potentially complex causal pathways.

As our analysis and a related analysis from Thomas and colleagues^15^ both identified strong evidence of an unadjusted association, but not of an adjusted association, between frailty and decisional regret, available data may suggest that a substantial proportion of the estimated unadjusted effect is meaningfully attributable to factors such as surgery type, age, sex, and mental health diagnoses. Although Thomas and colleagues^15^ analysed only head and neck surgery patients, our cohort that included multiple types of noncardiac surgeries provides further insights and supports generalisability. Importantly, surgery type appears to be a key factor to consider, as patients with frailty in our study having orthopaedic surgery had a lower likelihood of decisional regret than patients without frailty, whereas those living with frailty having non-orthopaedic surgery (mainly abdominal, vascular, and thoracic) had a higher probability of decisional regret than patients without frailty. Although our data do not provide direct insights into the mechanisms underlying this effect modification, differing recovery trajectories between surgery types may be explanatory. Specifically, patients having orthopaedic surgery likely experience improvements in physical function after surgery, whereas patients having abdominal and vascular surgery may not. Currently, the limited data available exploring long-term functional recovery after surgery for older adults with frailty is mixed. In noncardiac surgery, frailty is associated with improved disability scores at 1 year with no effect modification by surgery type.^42^ In cardiac surgery, frailty predicts poor long-term functional recovery, with effect modification by open *vs* minimally invasive approaches.^47^ Therefore, future research with long-term concurrent measurement of functional recovery and decisional regret will be required to provide deeper insights into these complex associations.

The interplay between frailty and related concepts such as comorbidity and disability are also complex and difficult to disentangle. For example, Fried and colleagues^35^ describe frailty, disability, and comorbidity as distinct entities. In contrast, the accumulating deficits model of frailty, from which our study's frailty exposure, the CFS, emerges, describes frailty as arising from comorbidity- and disability-related deficits (among others).^2^ Although epidemiologically, the choice of conceptual model directly informs the choice of confounders, and therefore strongly influences estimates of the association between frailty and decisional regret, for patients and clinicians, more practical actions from these data may be required. Although our data do not emerge from a randomised trial where causality can be directly inferred, patients and clinicians may wish to carefully consider comorbidity and disability as predictors of decisional regret along with frailty. However, regardless of the risk profile, clinicians should recognise that providing more information and ensuring meaningful involvement in the decision-making process are evidence-based approaches to reducing decisional regret after treatment decisions, such as having surgery.^7^

### Strengths and limitations

Our protocol was preregistered, which should reduce performance bias. Using a Bayesian framework, we provided estimates that may be more consistent with clinical reasoning, as our results reflect the probability of non-null associations and provide ranges of plausible true values for parameters given prior knowledge and study data. Although our interpretation of the strength of findings was informed by a 95% probability of non-null association representing a threshold, individual readers can interpret these probabilities as a continuum. Decisional regret was measured on an ordinal scale, which may be a strength as it provides greater information than simple dichotomous approaches. However, more information-rich measures such as the full Decision Regret Scale could provide a more detailed assignment of outcome status.^6^ We are ultimately unable to ascertain how much information might have been lost using a 3- *vs* 5-point Likert scale. A lack of clear reporting of decisional regret data in the orthopaedic literature may be a barrier to the contextualisation of our results.^8^^,^^48^ Although results may have differed with the use of a different frailty instrument, the CFS is an accurate and feasible tool that is guideline recommended.^49^ Similar to other outcomes, our results were numerically consistent with frailty assessed using the Fried phenotype.^5^^,^^15^ Lastly, although adverse outcomes after surgery could contribute to decisional regret, we did not adjust for such outcomes as they would act as mediators, not confounders, of the association under study.

### Conclusions

In a multicentre, prospective cohort study, older surgical patients living with frailty were more likely to report greater decisional regret across the year after surgery, but much of this effect was explained by patient and procedural factors. When supporting patients with frailty to make optimised, shared decisions about surgery, clinicians should consider frailty status in context with surgery type and a patient's comorbidity and disability status. Future prospective research is required to help disentangle the complex relationships between frailty, comorbidity, disability, surgical indication, and expected risk of decisional regret.

## Authors’ contributions

Conception: DIM, EH, YA, LB, LL, HM, ML

Study design: DIM, YA, EH, LB

Data acquisition: DIM, EH, ML, HM, LL

Data analysis: DIM, EH, YA, LB

Data interpretation: DIM, EH, YA, LB, LL, HM, ML

Drafting of the manuscript: DIM, YA, EH, LB

Revision of the manuscript: DIM, EH, YA, LB, LL, HM, ML

Approval of the final manuscript version: DIM, EH, YA, LB, LL, HM, ML

## Declaration of interest

The authors declare that they have no conflicts of interest.

## Funding

Peer-reviewed funding from the Canadian Frailty Network and The Ottawa Hospital Academic Medicine Organization; salary support from The Ottawa Hospital Anesthesia Alternate Funds Association and Clinical Research Chairs from the University of Ottawa, Faculty of Medicine (to DIM and MML). Mid-Career Knowledge Translation Award from the PSI Foundation (to DIM); Canadian Anesthesiologists' Society's Career Scientist Award (to MML). No funders played any role in planning, conduct, or reporting of results.
