# Supplementary material for: Frailty and decisional regret after elective noncardiac surgery: a multicentre prospective cohort study
Source: Br J Anaesth. 2024 Sep 3;133(5):965–72. doi: 10.1016/j.bja.2024.08.001 (PMC11488161; doi:10.1016/j.bja.2024.08.001)
Supplement: Multimedia component 1 [file mmc1.docx]

**Appendix 1 – Bayesian workflow, directed acyclic graphs and model diagnostics**

Weakly informative prior distribution (used for primary analysis): Student’s t, 3 degrees of freedom, scale=10

Informative prior distribution (used for sensitivity analysis to estimate sensitivity of results to the choice of prior distribution): Normal distribution with mean 0.69 on the log odds scale (equal to OR=2, a typical strength of association between frailty and postoperative morbidity and mortality) and SD=0.5.

Prior distribution for random effects: Half student-t prior with 3 degrees of freedom and a scale parameter dependent (minimum 2.5) on the standard deviation of the response after applying the link function (brms default).

**
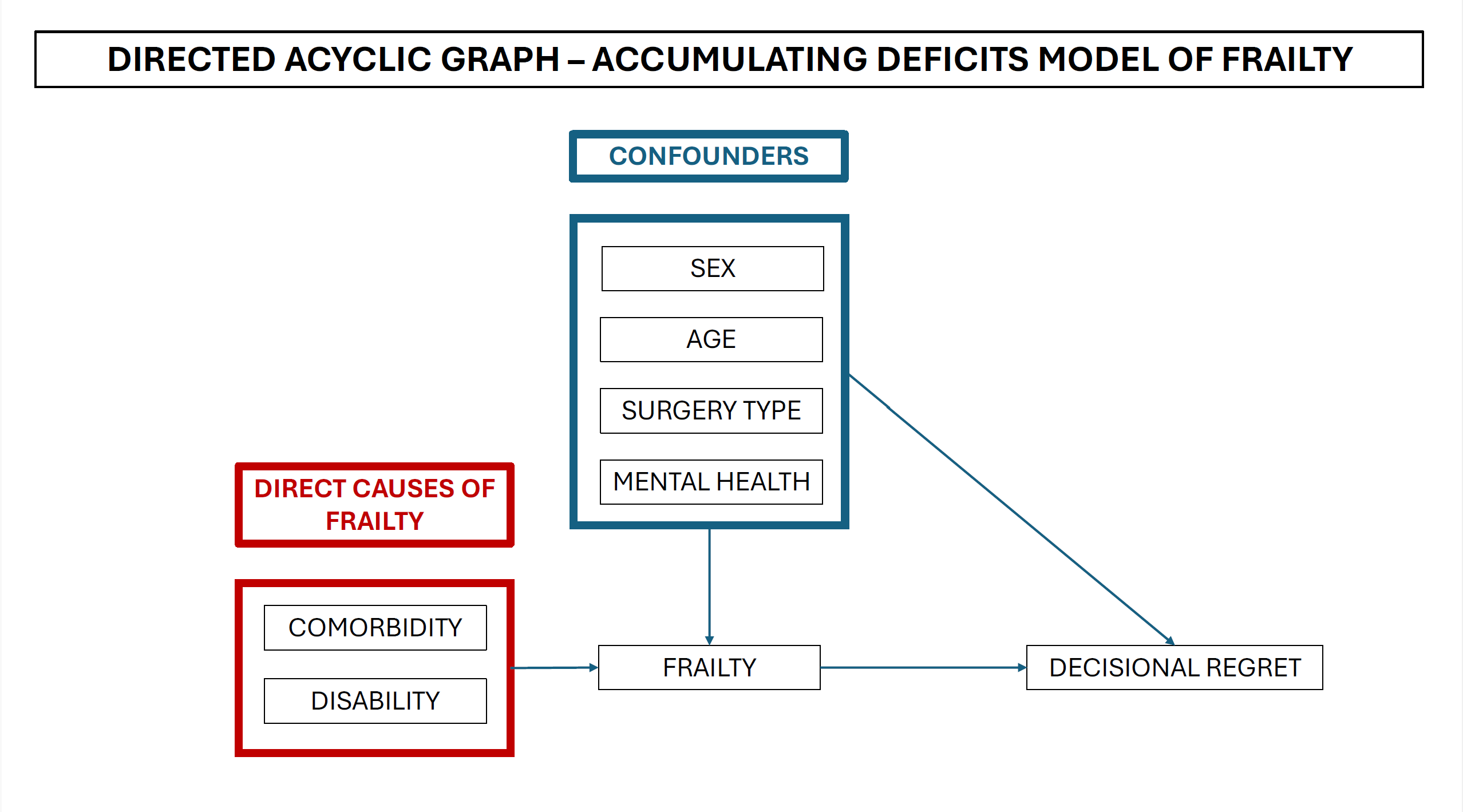
**

**
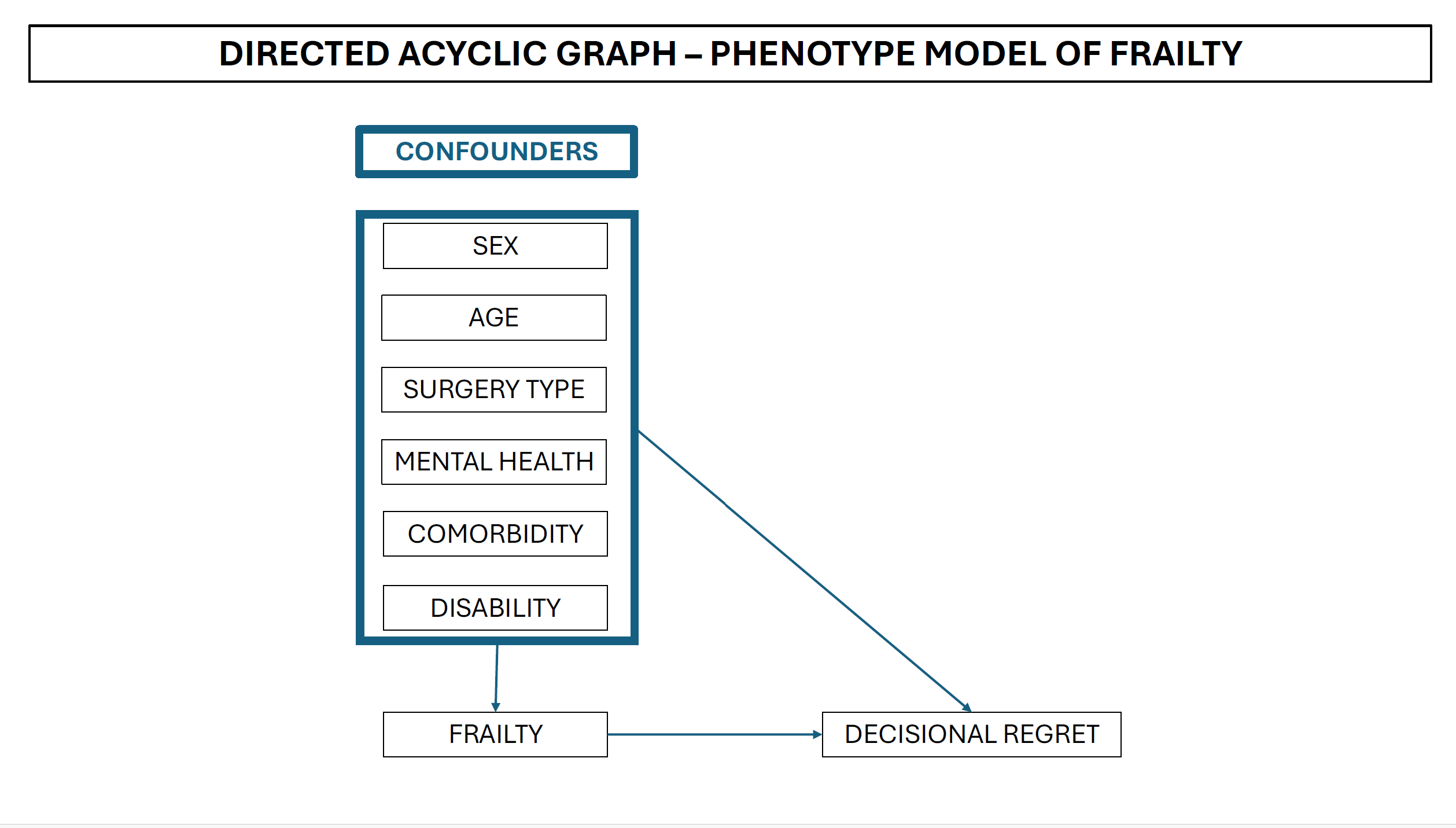
**

Iterations: 8000 total (warm up (4000); post-warm up (4000))

Control parameters: adapt_delta=0.99, max_treedepth=15

Model diagnostics for primary analysis (weakly informative prior):

There were no divergent transitions, and Rhat values for all parameters were <1.01

Trace plots and autocorrelation plots for each parameter are provided below. All autocorrelation plots suggested adequate mixing of chains, while plots of the distribution of sampled parameters were all unimodal and normally distributed, suggesting good model fit and exploration of the parameter space:

| 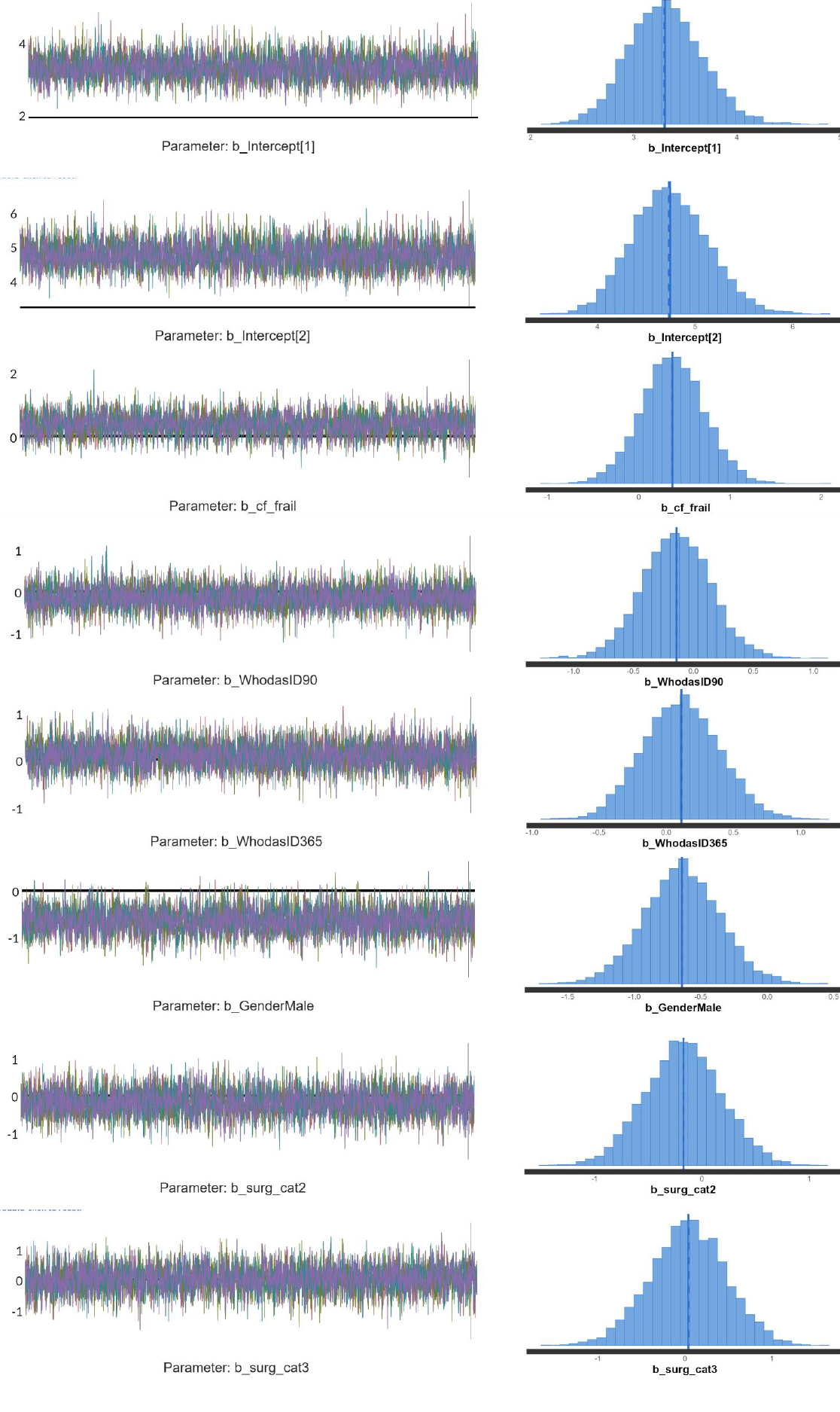 | 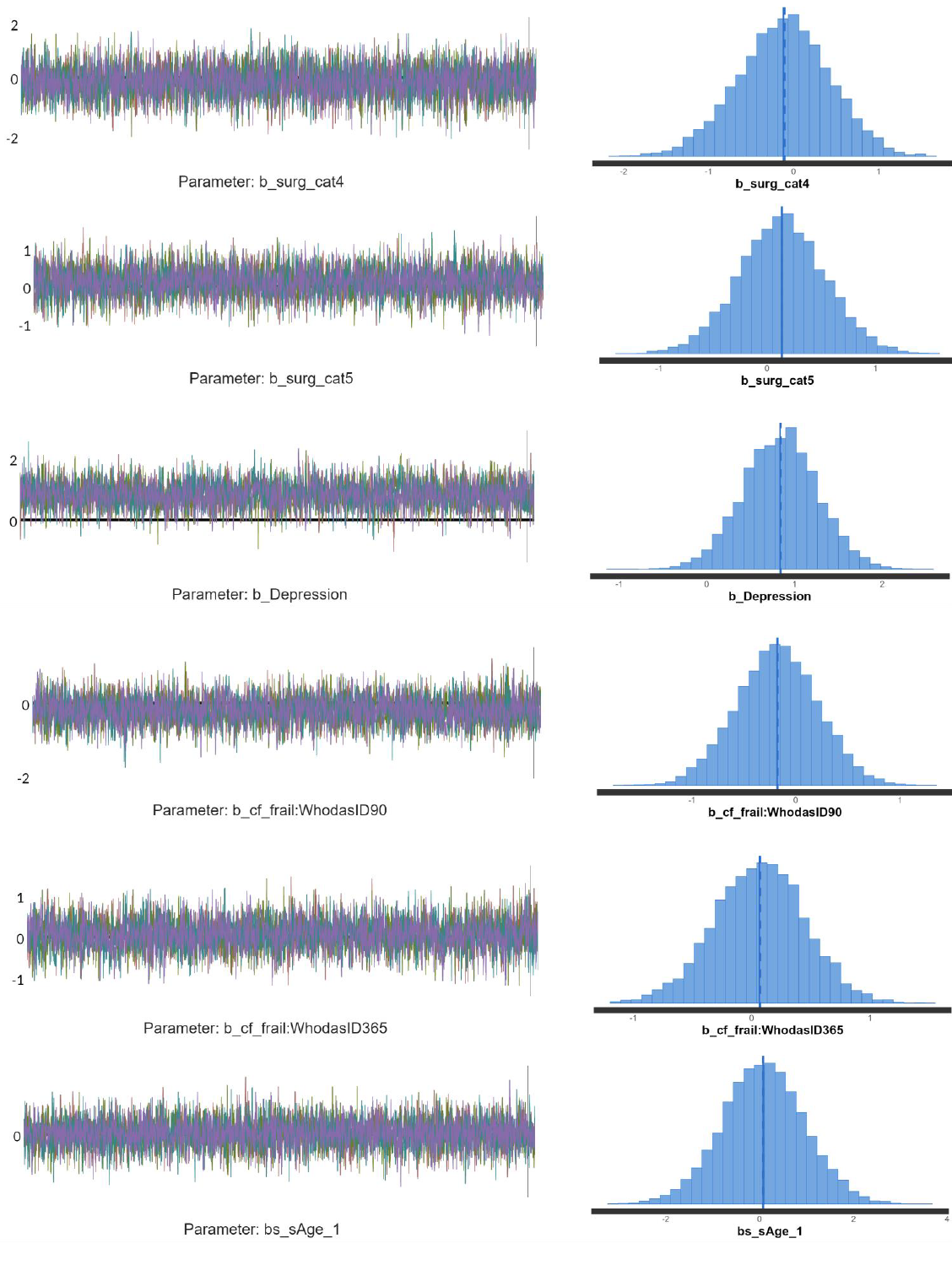 |
| --- | --- |

**Appendix 2 – Multiple imputation and missing data**

We imputed 5 complete datasets under a missing at random assumption using the ‘mice’ package in R. Decisional regret severity was imputed using predictive mean matching.

The following variables were used in the multiple imputation model: cognitive dysfunction (binary), anxiety or depression risk (binary), Elixhauser comorbidity index (categorical), Clinical Frailty Scale Score (categorical), baseline/30/90/365 day disability score (continuous), 30/90/365 day mortality status (binary), type of surgery (categorical), study site (categorical), 30/90/365 day decisional regret status (ordinal), sex (binary), age (continuous).

Unlike analysis of imputed datasets under frequentist frameworks, which combine analyses of each data set and compute central tendency and variance using statistical rules (i.e., Rubin’s Rules), estimation of parameters of interest from imputed results in Bayesian analyses involves pooling posterior distributions from all analyses of imputed data sets, followed by calculation of parameters of interest (in our case the OR, 95CrI and P(OR>1)) from the pooled posteriors. In other words, final results are directly calculated from the pooled posterior across imputations.

Baseline characteristics of participants with vs without missing outcome data are provided below:

**Participant characteristics by missing data status**

|  | **Missing Data** | | **ASD** |
| --- | --- | --- | --- |
|  | **No (n=579)** | **Yes (n=90)** |  |
| **Demographics** |  |  |  |
| Age (years), mean (SD) | 73.5 (6.2) | 74.8 (6.0) | 0.21 |
| Comorbidity, mean (SD) | 2.2 (1.5) | 2.7(1.7) | 0.31 |
| Disability, mean (SD) | 21.7 (16.3) | 22.5 (19.0) | 0.05 |
| Female (%) | 48.6 | 43.2 | 0.11 |
| Cognitive dysfunction (%) | 35.4 | 41.1 | 0.12 |
| Depression (%) | 7.8 | 10.2 | 0.08 |
| **Surgery (%)** |  |  |  |
| Abdominal | 19.7 | 31.1 | 0.26 |
| Neurologic | 5.5 | 5.6 | 0 |
| Orthopedic | 50.8 | 34.4 | 0.34 |
| Thoracic | 9.7 | 13.3 | 0.11 |
| Vascular | 14.3 | 15.6 | 0.04 |

**Participant and missing data flow**


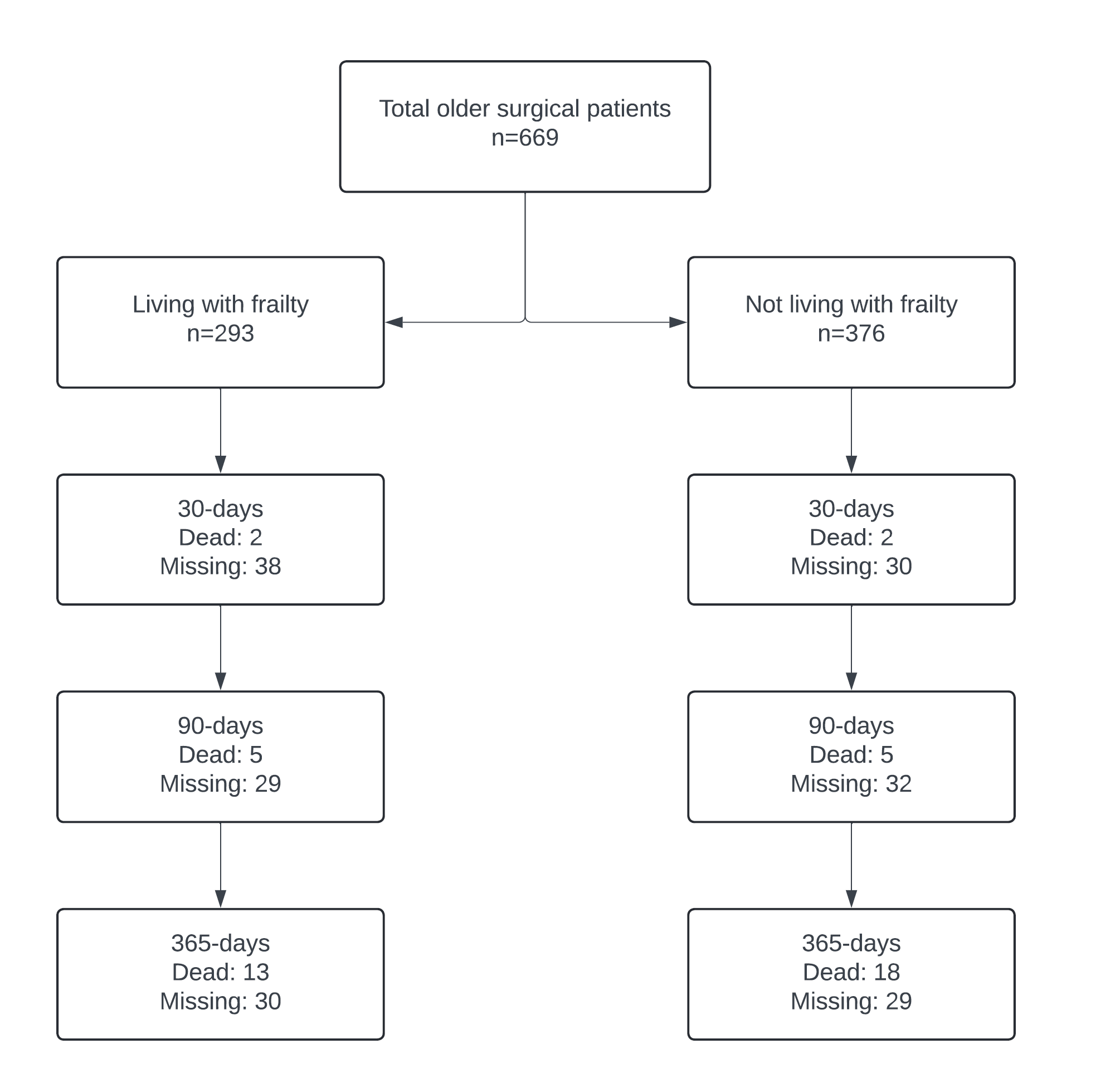


**Appendix 3 – Ordinal decisional regret responses by time and frailty status**

| **Time** | **Exposure** | **No** | **Unsure** | **Yes** |
| --- | --- | --- | --- | --- |
| 30-days | Without frailty | 0.92 | 0.05 | 0.04 |
| 30-days | With frailty | 0.87 | 0.08 | 0.05 |
| 90-days | Without frailty | 0.92 | 0.06 | 0.02 |
| 90-days | With frailty | 0.90 | 0.07 | 0.03 |
| 365-days | Without frailty | 0.91 | 0.05 | 0.04 |
| 365-days | With frailty | 0.86 | 0.08 | 0.06 |

Note: Values under columns No, Unsure, Yes represent the proportion of respondents providing each ordinal value as a response. Proportions are rounded to the 2^nd^ decimal place, therefore rounding may lead to total proportions >1.00

**Appendix 4 – Results of primary adjusted analysis under informative priors**

| Day | OR | 95%CrI(L) | 95%CrI(U) | *P*(OR>1) |
| --- | --- | --- | --- | --- |
| 30 | 1.60 | 0.91 | 2.82 | 0.94 |
| 90 | 1.35 | 0.67 | 2.7 | 0.80 |
| **365** | 1.61 | 0.81 | 3.12 | 0.91 |

OR: odds ratio; 95%CrI: 95% credible interval (L) lower and (U) upper; *P*(OR>1):probability that the true odds ratio is greater than one given the prior distribution and the data.
