# Supplementary material for: Frailty and decisional regret after elective noncardiac surgery: a multicentre prospective cohort study
Source: Br J Anaesth. 2024 Sep 3;133(5):965–72. doi: 10.1016/j.bja.2024.08.001 (PMC11488161; doi:10.1016/j.bja.2024.08.001)
Supplement: Multimedia component 2 [file mmc2.docx]

| **The WAMBS-Checklist**When to worry, and how to Avoid the Misuse of Bayesian Statistics **Depaoli & Van de Schoot (2016)** | | | |
| --- | --- | --- | --- |
|  | **Location** | **Criterion met** |  |
| **To be checked before estimating the model** |  |  |  |
| **Point 1:** Do you understand the priors? | Appendix 1 | YES |  |
| **To be checked after estimation but before inspecting model results** |  |  |  |
| **Point 2:** Does the trace-plot exhibit convergence? | Appendix 1 | YES |  |
| **Point 3:** Does convergence remain after doubling the number of iterations? | Appendix 1 | YES |  |
| **Point 4:** Does the histogram have enough information? | Appendix 1 | YES |  |
| **Point 5:** Do the chains exhibit a strong degree of autocorrelation? | Appendix 1 | NO |  |
| **Point 6:** Does the posterior distribution make substantive sense? | Appendix 1 | YES |  |
| **Understanding the exact influence of the priors** |  |  |  |
| **Point 7:** Do different specifications of the multivariate variance priors influence the results? | Appendix 1& 3 | NO |  |
| **Point 8:** Is there a notable effect of the prior when compared with non-informative priors? | Appendix 1 & 3 | No |  |
| **Point 9:** Are the results stable from a sensitivity analysis? | Results, Figures | Yes |  |
| **After interpretation of model results** |  |  |  |
| **Point 10**: Is the Bayesian way of interpreting and reporting model results used? *(a) Also report on: missing data, model fit and comparison, non-response, generalizability, ability to replicate, etc.* | Results, Discussion | YES |  |
